# The role of numerical magnitude and order in the illusory perception of size and brightness

**DOI:** 10.3389/fpsyg.2013.00484

**Published:** 2013-07-29

**Authors:** Arnaud Viarouge, Maria Dolores de Hevia

**Affiliations:** ^1^Laboratoire Psychologie de la Perception, UMR 8158 CNRS, Université Paris DescartesParis, France; ^2^INSERM U992, NeuroSpin, CEA, DSV/I2BMGif/Yvette, France

**Keywords:** numerical cognition, cognitive Illusion, magnitude system, brightness perception

## Abstract

Processing magnitudes constitutes a common experience across multiple dimensions, for example when one has to compare sizes, duration, numbers, sound height or loudness. From a cognitive point of view, however, it is still unclear whether all these experiences rely on a common system, or on distinct systems, with more or less strong associations. One particularly striking way of observing such interference between the spatial and numerical dimensions consists in eliciting a bias in size judgment through the mere perception of irrelevant numerical stimuli. In such experimental context though, two questions remain open. First, it is still unknown whether the direction of the bias is related to the magnitude of the number presented, or to their position in an ordinal sequence, and thus could be elicited by other non-numerical ordinal sequences such as letters of the alphabet. Second, it is still unclear whether the observed interactions generalize to other continuous dimension of magnitude such as brightness. In the study reported here, both letters and numbers were used in a size- and a brightness-reproduction task. We observed a dissociation between the two types of stimuli when reproducing size, the illusion being elicited solely by numbers. When reproducing brightness, however, neither the letters nor the numbers elicited a bias. These findings suggest that, while only numerical magnitude, and not letters, elicits a bias in size perception, the concurrent processing of magnitude and brightness does not bring about the same illusion, supporting the idea of a relative independence in the processing of these two dimensions.

## Introduction

A number of studies in the field of numerical cognition converge toward the idea that numerical representations are rooted in intuitions about fundamental dimensions of our perceptual world, such as space and time. In particular, numbers and space appear to be intimately related in the human mind. This idea has been supported by various behavioral and neuroimaging studies (Galton, 1880; Restle, 1970; Dehaene, 1992; Pinel et al., 2004; Hubbard et al., 2005). The neuroimaging studies notably point toward shared neural resources for numerical and spatial cognition, by showing an overlap between the activated areas involved in each process, in particular in the intra-parietal region (Fias et al., 2001; Dehaene et al., 2003; Pinel et al., 2004). On these grounds, some researchers have postulated the existence of a single system for the representation of magnitude that is shared across all continuous dimensions (Walsh, 2003; Bueti and Walsh, 2009). However, others have proposed the existence of distinct representations sharing more or less strong associations (Pinel et al., 2004), with space and number possibly sharing a privileged link (de Hevia et al., 2012). The present study aims at contributing to this debate by investigating whether numbers can affect the representation of a non-spatial continuous dimension, i.e., level of brightness. Moreover, we also investigated the specific role of numerical magnitude, as opposed to ordinality, in eliciting these interactions between continuous dimensions. In fact, general processing of ordinality has shown to recruit neural resources in the intra-parietal region that are shared by numbers and other non-numerical sequences, such as letters of the alphabet (Fias et al., 2007).

The most well-known example of number-space association is the SNARC effect (Spatial-Numerical Association of Response Codes; Dehaene et al., 1993), which consists of a reaction time advantage in numerical tasks when the side of response is compatible with the size of the number (large numbers responded faster on the right-sided response button, small numbers responded faster on the left-sided response button). This effect reflects the automatic association between numbers and oriented spatial codes, as it has been observed even in experimental contexts where the numerical magnitude is irrelevant to the task at hand (such as in parity judgments) (Dehaene et al., 1993; Fias et al., 1996; Fias, 2001). Other experimental paradigms have provided evidence of an association of numbers to oriented spatial codes, such as the lateralized shifts of attention brought about by numerical magnitude (Fischer et al., 2003), spontaneous lateralized actions (key presses, Daar and Pratt, 2008; or random finger movements, Vicario, 2012) and SNARC-like effects related to saccadic eye movements (Fischer et al., 2004; Loetscher et al., 2010). It is still unclear, however, at what level of cognitive processing these number-space interactions take place, with accounts ranging from an early interference at the representational level (Walsh, 2003) to an interference at a late, response-related, stage of processing (Keus and Schwarz, 2005; Gevers et al., 2006; but see also Koten et al., 2011 for an account including both early and late stage of processing).

While the SNARC and related effects can be taken as evidence of an association between numerical magnitudes and corresponding spatial positions, other studies have investigated the interaction between numbers and spatial extents, without implying any directionality or information about spatial position. For instance, in a numerical Stroop task where participants have to compare two visually presented digits based either on their numerical magnitude or their physical size, eventual interference and/or disruptive effects are measured. Through this paradigm, the physical size of the two digits in the pair can be either congruent, in which case both participants' accuracy and reaction times are improved, or incongruent, leading to impaired accuracy and longer reaction times (see Henik and Tzelgov, 1982; Girelli et al., 2000; Pinel et al., 2004). Notably, the interference/facilitation effects take place even though only one magnitude (physical or numerical) is relevant during one given task (size or number comparison). These findings therefore suggest that the mere presentation of a numerical stimulus is sufficient to activate the corresponding representation of its magnitude, which in turn interferes with the processing of magnitude in the other dimension.

The paradigm of the cued line bisection task has also been exploited to investigate the interaction between numerical and spatial cognition (see Fischer, 2001a for a review). While some studies report SNARC-like effects in the bisection of numerical strings that are interpretable on the basis of lateralized spatial codes, (i.e., a leftward bias in the bisection of stimuli made of small numbers, e.g., 111111111111, and a rightward bias for large numbers, e.g., 99999999999999; Fischer, 2001b; Calabria and Rossetti, 2005; see Bonato et al., 2008 for a study in patients with spatial neglect), another series of studies have interpreted the spatial biases related to numbers as evidence of an influence of numerical information on the representation of spatial extent. In the experimental context of a bisection task with a line flanked by two different digits (e.g., 1—-8), a bias toward the larger digit is observed, irrespective of its spatial position (de Hevia et al., 2006; de Hevia and Spelke, 2009; Ranzini and Girelli, 2012). This effect was interpreted as reflecting an illusion of lateral disparity of the spatial extension induced by the magnitude of the flanker (illusion of larger extent on the side of the larger numerical flanker, de Hevia et al., 2006). Speaking even more strongly for an illusion of spatial extent elicited by numerical magnitude, when participants reproduce the spatial extent delimited by two identical digits in a computerized reproduction task, by symmetrically adjusting the space so that no lateralized effects could be observed, a systematic overestimation was reported for large numbers (e.g., 8 8 and 9 9), relative to small numbers (e.g., 1 1 and 2 2; de Hevia et al., 2008). Overall, these findings have been interpreted as reflecting the existence of an “illusory” perception of a larger/smaller spatial extent conveyed by larger/smaller digits, respectively (de Hevia et al., 2006, 2008).

Thus, there is a large amount of evidence showing a variety of interactions between numbers and space, suggesting they share a privileged relationship. The studies described above show that this relationship can either consist in an interaction between numbers and spatial location or spatial extent. Spatial extent being more readily conceived in terms of a continuous dimension, we chose to focus on its relationship with numerical magnitude in the present study.

### The role of order in magnitude interactions

In the case of numbers, information regarding both magnitude and ordinality can be conveyed by a numerical stimulus. To date, the question of the exact role of magnitude vs. ordinality information contained in numerical stimuli in eliciting a number-space interference is still strongly debated (see for instance Fias et al., 2007). However, it seems that the question of whether other information contained in the stimulus could explain the interference observed is crucial when assessing the unicity or plurality of representational systems for magnitude. Previous studies have shown that the position of numbers in an ordinal sequence could be what triggers the number-space association in the SNARC effect. In fact, a SNARC-like effect has been observed with respect to the order of items belonging to non-numerical ordinal sequences such as letters or days of the week (Gevers et al., 2003, 2004), tone height (Rusconi et al., 2006), past and future events (Santiago et al., 2007), and even with unrelated words memorized in a list (Previtali et al., 2010). While the SNARC effect has been interpreted by several authors as supporting the thesis of a spatial representation of numbers, other authors have argued that invoking the spatial nature of numerical representation is unnecessary to account for the number-space interaction observed in these studies (Santens and Gevers, 2008; Gevers et al., 2010; Van Dijck and Fias, 2011). For instance, Van Dijck and Fias (2011) postulate that the automatic association of the order of numerical stimuli in working memory with spatial positions is responsible for the observed number-space association. Independently of the spatial nature of the numerical representation, both accounts involve the spatial ordering of numbers (either at the representational level, or as spatial positions in working memory), which could explain why the role of the ordinal and cardinal aspects of numbers is difficult to disentangle in this type of paradigm. However, some studies have shown that numerical magnitude is crucial to observe spatial biases in specific experimental contexts (Casarotti et al., 2007; Perrone et al., 2010). For example, Casarotti et al. (2007) show that numbers, and not letters, can elicit a bias in spatial attention, resulting in a bias in the perception of the temporal order of laterally presented visual stimuli.

Moreover, in their study, Ranzini and Girelli (2012) observed a modulation of the bias in the purely numerical version of the cued line bisection task, depending on the numerical distance between the digits (but see de Hevia et al., 2006 for absence of this effect using a similar paradigm). The distance effect has been previously observed with stimuli from ordinal sequences (Jou and Aldridge, 1999), at least in the context of tasks requiring a comparison process (Van Opstal et al., 2008). If one assumes that the distance effect reflects the activation of an ordered representation of numbers, the observed modulation by numerical distance in (Ranzini and Girelli, 2012) would make it possible that the ordinal aspect of numbers plays a role in the bias observed when bisecting lines flanked with distinct digits.

Thus, the first goal of our study was to investigate the exact role of magnitude vs. ordinality in an experimental context known to elicit interference between numbers and spatial extent, such as the size reproduction task (de Hevia et al., 2008).

### The link between numbers and brightness

The second issue we investigated in the present study was whether the kind of illusion observed in the reproduction task generalized to other magnitude dimensions, such as the brightness level. Walsh (2003) postulated in his ATOM (A Theory of Magnitude) theory the existence of a single system for the representation of magnitude that is shared across all continuous dimensions (see also Bueti and Walsh, 2009 for an updated version including recent supportive evidence from neuroimaging studies, where the authors are also cautious in stating that all dimension should behave identically, both at the behavioral and neural level). However, others have proposed the existence of distinct representations sharing more or less strong associations (Pinel et al., 2004), with space and number possibly sharing a privileged link (de Hevia et al., 2012).

The dimension of brightness is known to interfere with the representation of other perceptual dimensions such as size or time duration. For instance, brighter stimuli are perceived as lasting longer (Xuan et al., 2007), and differences in brightness affect perception of size (Westheimer, 2008; Walker and Walker, 2012). A recent study observed SNARC-like effects when participants had to perform size, luminance and conceptual size comparison tasks with lateralized responses (Ren et al., 2011). These results are in agreement with one prediction of the ATOM theory, the generalization of the SNARC effect to what Walsh refers to as the SQUARC (spatial quantity association of response codes), according to which non-numerical magnitude could also elicit similar response compatibility effects with space. Other studies have shown the possible interference between numbers and brightness, notably in the context of comparative judgments when varying the congruency between the two dimensions, although the evidence for mutual interference between the two dimensions is still weak Pinel et al., 2004; Cohen Kadosh and Henik, 2006; Cohen Kadosh et al., 2008a. Pinel and colleagues (2004), as well as Cohen Kadosh and Henik (2006), observed that incongruence between brightness and numbers was impacting reaction times during a number comparison task. However, the symmetrical interference (impact of number on brightness judgments) was only observed in the latter study, whereas in the former study a symmetrical pattern of interference was found only between numbers and size, and brightness and size. Regarding the neural correlates of these interferences, again, the results are diverging, the brightness-number interference modulating brain activity (in the right IPS) only in the aforementioned study by Cohen Kadosh et al. (2008a).

In a recent study, Ranzini and Girelli (2012) observed a bias in a line bisection task using distinct square flankers which differed in luminance. In addition to replicating the previously found bias toward the larger of two distinct numerical flankers, the authors observed a similar, although less pronounced bias toward the darker (or the one producing the higher contrast) of the two square flankers. Interestingly, they also observed a modulation of the bias when using numerical flankers with a luminance either congruent or incongruent with the numerical magnitude, showing a possible interference between the visual illusion elicited by brightness and the cognitive illusion elicited by numbers. The authors interpreted the latter result as supporting the view of distinct but overlapping representations of magnitude across physical and numerical dimensions (see also Pinel et al., 2004).

Thus, the second goal of our study was to further explore the possible link between number and brightness by adapting the reproduction task for the brightness dimension. This adapted version of the task allowed us to test for the existence of a similar illusory perception of brightness brought about numbers and/or letters of the alphabet, and thus assess the possibility of a common underlying representational system of magnitude accounting for these cognitive illusions between numbers, spatial extents and brightness levels. Indeed, if one postulates the existence of shared systems to represent and/or compare magnitudes across all continuous dimensions, then the illusion observed in the reproduction task should also be found when asking the participants to reproduce the stimulus based on properties other than size, such as brightness. Alternatively, the existence of a shared representational system or a strong cognitive link for magnitudes could be limited to dimensions such as space, time and number, and associations with other continuous dimensions such as brightness could be weaker or more indirect.

In summary, our study was aimed at better understanding the exact role of numerical information in its interaction with other continuous dimensions. On one hand, we wanted to assess the respective contribution of numerical magnitude and ordinality in these interactions. On the other hand, we wanted to test for a possible generalization of these interactions to the brightness dimension. More specifically, we investigated whether ordinality alone could account for the cognitive illusion observed in a reproduction task (de Hevia et al., 2008). If the (irrelevant) magnitude information in the numerical stimulus is crucial to elicit a cognitive illusion of spatial extent, then this illusory effect should not be found when using flankers belonging to a non-numerical ordinal sequence, such as letters of the alphabet, because they convey no information relative to magnitude. Moreover, we asked whether numerical magnitude and/or ordinal information might also elicit an illusory perception of a non-spatial continuous dimension, i.e., brightness. A shared representation of magnitude should result in a similar cognitive illusion between numbers and brightness.

## Materials and methods

In the experiment reported here, participants were first presented with a square surrounded by symbols (either letters or numbers). They were then showed another square and asked to modify its size or brightness in order to reproduce the size or brightness of the first presented square.

### Participants

Twenty-five participants took part in this experiment. Two participants' data were removed based on a difference in estimation exceeding the average ±2.5 standard deviation range. Hence, the data reported here are based on the remaining 23 participants (10 male, 13 female, mean age = 27.61, *sd* = 4.49). All participants were naïve to the hypotheses of this study.

### Stimuli and procedure

The experiment was programmed using the Matlab software and the Psychtoolbox library (Brainard, 1997), and was run on a 64 bits dell laptop (2.9 GHz processor). The stimuli were presented on a 15.6″ screen with a resolution of 1920 × 1080 pixels and a refresh rate of 60 Hz. The task was divided in four blocks. The participants were asked to perform a brightness-reproduction task during two blocks, and a size-reproduction task during two other blocks. The order of the four blocks followed a latin square design, leading to four possible orders. Each order was run by six of the 23 participants reported here, except one order run by 5 participants.

**Figure 1:**
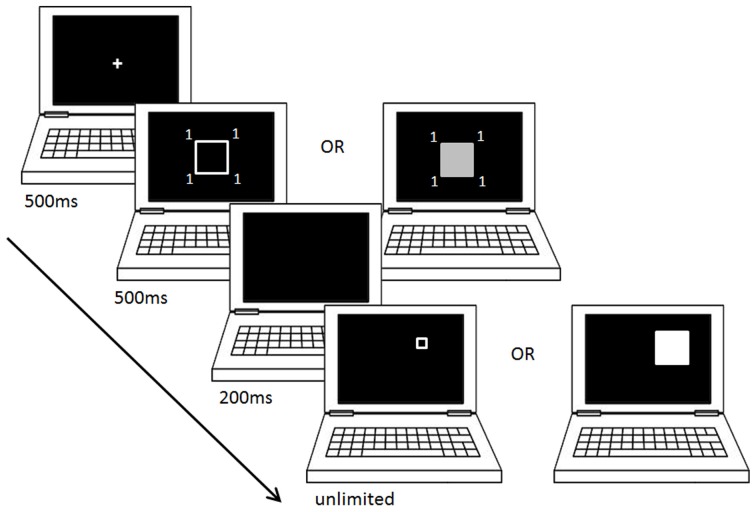
**Size and brightness reproduction tasks**. This figure shows the experimental design of the size- and the brightness-reproduction task, for one trial of a block where the first presented square is flanked with a small digit.

In both the brightness and the size reproduction tasks, the design was as follows (all stimuli presented on a black background): after presentation of a fixation cross at the center of the screen for a duration of 500 ms, a first square was presented for 500 ms, also centered on the screen, and flanked with four symbols outside of each corner. Then the first square disappeared for 200 ms, and a second square (the target) was presented, centered on one of two possible locations on the screen (either toward the top-left or the top-right of the screen). The participants had then an unlimited amount of time to modify the target square, using the “1” (smaller/darker) and “3” (larger/brighter) keys on the numerical keypad, and press the “5” key to validate their response when they felt like the target square matched the first presented square. A blank screen was then presented for 1 s before the next trial (Figure 1).

For each reproduction task (size or brightness), the symbols were letters in one block, and numbers in the other block. Four numbers and four letters were used. They were chosen so that, in each category, two items would belong to the beginning of an ordinal sequence (1, 2 and A, B), and two items would belong to the end of an ordinal sequence (8, 9, and Y, Z). Contrarily to the four letters which only convey the information about ordinality described above, the four numbers chosen also convey an information relative to numerical magnitude (two small and two large numbers, relative to the numerical range used in this study). The symbols were presented in Arial font, size 32 (representing a surface of 7 × 4 mm^2^), at a distance of approximately 1 mm from each corner on the diagonal axes of the square. The screen was maintained at a distance of approximately 50 cm from the participants' eyes.

In the size reproduction task, the first square was an unfilled white square (line thickness = 1 mm), which could be of three different sizes (area of 31 × 31, 46 × 46, or 61 × 61 mm^2^). The starting size of the second square could be either very small (5 × 5 mm^2^), or very large (86 × 86 mm^2^), and the step size for each key press was approximately 0.5 mm. In the brightness reproduction task, the first square was a filled square with an area of 46 × 46 mm^2^ which brightness could vary according to three different levels corresponding to three shades of gray (identical rgb values = 75, 125, or 175). Since all the subjects ran the experiment on the same screen in the same lighting conditions, we use the rgb values as our index of brightness in the rest of the manuscript. The second square had an identical size as the first one, and the starting rgb value could be either very low (25) or very high (225). The step size for each key press corresponded to an rgb difference of 1. In both tasks, the side of presentation of the target square was counterbalanced between trials, and so were the three levels of brightness or size (depending on the task) for the first square. A maintained key press by the participants resulted in a continuous change in the considered dimension. Subjects were asked to reproduce, as accurately as possible and without time limits, the size or brightness level of the first square presented. The instructions mentioned that the first square would be surrounded by fours symbols, which were unrelated to, and did not predict, either the size or the brightness level of the first square.

For each task, participants completed a total of 96 trials (one trial per condition), with a total duration of approximately 20 min. For each trial, depending on the block, the error of estimation [estimated size (mm^2^) − actual size (mm^2^) or estimated brightness (rgb value) − actual brightness (rgb value)] was computed.

## Results

First, trials showing an estimation error outside the mean ±2.5 *sd* for each participant were removed (which corresponded to less than 4 trials for each participant in each dimension). For each condition (type of symbol × level of ordinality × starting size/brightness), we then averaged the estimation error across trials. In order to compare the data from the size-reproduction task (in mm^2^) and the brightness-reproduction task (rgb value), and avoid observing a task effect that would be an artifact of the scale, we standardized the data within each task by dividing the computed average estimation error for each subject and each condition by the standard deviation across the corresponding task. We then ran a 2 (tasks) × 2 (letters vs. numbers) × 2 (beginning vs. end of the ordinal sequence) × 2 (starting size/brightness of the second square) repeated measures ANOVA on the standardized average estimation error.

We observed a main effect of task [*F*_(1, 22)_ = 16.87, *p* < 0.001] showing that estimation error was larger in the size- than in the brightness-reproduction task (0.46, corresponding to an overall overestimation of 167.53 mm^2^ vs. −0.10, corresponding to an overall underestimation of -2.45 in rgb value). We also observed a main effect of starting size/brightness [*F*_(1, 22)_ = 13.13, *p* = 0.001] that appeared to be driven by a starting size effect in the brightness-reproduction task, as demonstrated by a significant task × starting size/brightness interaction [*F*_(1, 22)_ = 25.92, *p* < 0.001]. This interaction consisted in a larger overestimation in the brightness-reproduction task for the brighter starting value (0.64, i.e., an overestimation of 13.94 in rgb value), and underestimation for the darker starting value (−0.86, corresponding to −18.73 in rgb value), whereas a global overestimation was found in the size-reproduction task (0.43 for small starting size corresponding to 158.79 mm^2^ vs. 0.48 for large starting size corresponding to 176.22 mm^2^). LSD *post-hoc* tests confirmed the presence of an effect of the starting value in the brightness task (*p* < 0.001), and an absence of such effect in the size task (*p* = 0.84). This effect of the starting value specifically in the brightness-reproduction task could be reflecting the reliance on the anchor point, which might significantly affect brightness memory (see Uchikawa and Ikeda, 1986 for evidence of poor performance in comparing brightness levels simultaneously presented). Importantly, for the rest of the results described below the starting value did not appear to interact with either the type of symbol used or the ordinal position.

More crucially for the current study, we observed an interaction between symbol (numbers vs. letters) and the position in the ordinal sequence (1, 2, A, B vs. 8, 9, Y, Z) [*F*_(1, 22)_ = 7.07, *p* = 0.01]. A significant triple interaction between task, symbol and position in the ordinal sequence was also found [*F*_(1, 22)_ = 6.32, *p* = 0.02] allowing us to perform *post-hoc* tests in order to investigate the interaction between symbols and ordinal position within each task separately, as described below.

### Size reproduction task

**Figure 2:**
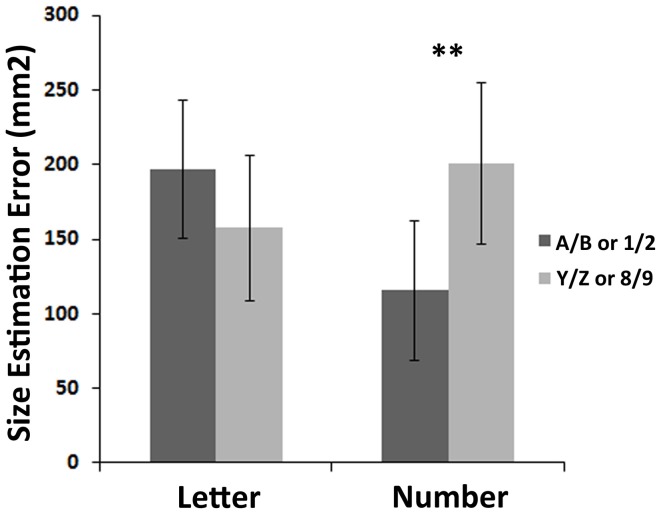
**Results of the size reproduction task**. This graph shows the bias in size (in mm^2^) elicited in the size reproduction task when using letters or numbers as irrelevant flankers, for “small” (A/B or 1/2) vs. “large” (Y/Z or 8/9) symbols. Double asterisks indicate a significant *post-hoc* test between overestimation for large and small numbers (*p* = 0.004).

In the size reproduction task, LSD *post-hoc* tests showed an effect of numerical magnitude, with a larger overestimation for large numbers than for small numbers (0.54 i.e., 200.66 mm^2^ vs. 0.32 i.e., 115.8 mm^2^, *p* = 0.004), in agreement with previous studies (de Hevia et al., 2008). However, no such effect was found with letters (overestimation of 0.54, i.e., 196.52 mm^2^ for letters A and B vs. 0.43, i.e., 157.66 mm^2^ for letters Y and Z, *p* = 0.15; Figure 2).

### Brightness reproduction task

**Figure 3:**
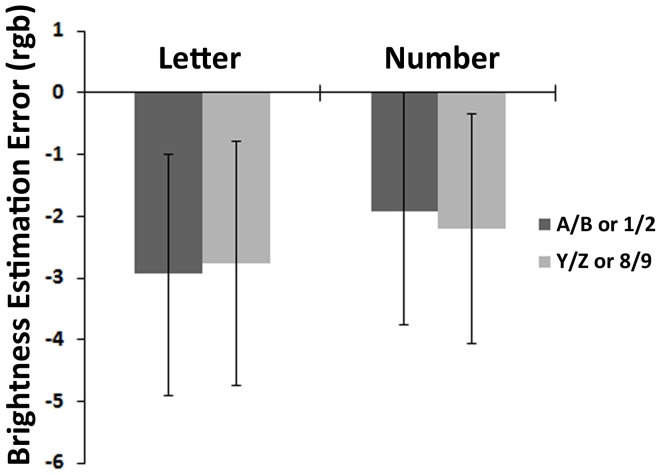
**Results of the brightness reproduction task**. This graph shows the bias in brightness (rgb value) elicited in the brightness reproduction task when using letters or numbers as irrelevant flankers, for “small” (A/B or 1/2) vs. “large” (Y/Z or 8/9) symbols.

In the brightness reproduction task, LSD *post-hoc* tests revealed no significant estimation bias in any of the two symbol types (letters: −0.13, i.e., −2.93 in rgb value for A, B vs. −0.12, i.e. −2.77 in rgb value for Y, Z, *p* = 0.93, and numbers: −0.08, i.e., −1.91 in rgb value for 1, 2 vs. −0.09, i.e., −2.2 in rgb value for 8, 9, *p* = 0.81; Figure 3).

## Discussion

In this study, we investigated the effects of illusory perception elicited by numerical and non-numerical symbols in a reproduction task. First, using these two distinct categories of symbols (numbers and letters), we could assess whether the cognitive illusion was solely due to the ordinal aspect of the stimuli used, or whether this task was indeed reflecting interference between quantity and other dimensions of magnitude. Second, we investigated the possible extension of the same cognitive illusion to the perception of brightness, which is another continuous dimension of magnitude.

Our results show that, when participants are asked to reproduce a square flanked by symbols belonging to an ordinal sequence, the interaction between symbol type (number vs. letter) and magnitude/ordinal position impacts their behavior differently depending on the continuous dimension they are asked to reproduce (spatial extent vs. brightness). In the context of a size reproduction task first, we replicated previous results showing that the presence of irrelevant digits surrounding the stimulus to be reproduced led to a cognitive illusion of spatial extent (de Hevia et al., 2008). The reported bias in estimation could be due to an illusory perception of the total surface, or result from the cumulative illusory perceptions of the sides of the presented square. On average, participants showed a larger overestimation of the spatial extent when the square was surrounded with the digits 8 or 9, relative to when those digits were 1 or 2.

In the same task, we show that non-numerical ordinal sequences such as letters do not elicit the same kind of cognitive illusion. In particular, when the first presented square was flanked with four letters, there was no bias depending on the position of the letters in the ordinal sequence of the alphabet (e.g., “A” vs. “Z”). This finding supports the idea of the specificity of numerical magnitude in eliciting the bias in perception of spatial extent observed in that experimental context. Although the continuous dimensions of number, size and brightness tested here are intrinsically ordered, this ordinal aspect alone does not appear to elicit the interference studied here.

In contrast with these results, it has been previously shown that letters can elicit a SNARC-like effect, even in the context of an order-irrelevant task (Gevers et al., 2003, 2004), suggesting that the association between number and spatial position observed in such paradigms is unlikely to rely solely on representations of continuous magnitude. It is worth noting though that other SNARC-like effects such as cued shifts of attention were found to be strictly elicited by numbers, at least when the order did not have to be explicitly processed (Dodd et al., 2008; Perrone et al., 2010). In experimental contexts when the participants had to process the order of the cue, the authors found that letters could elicit such a shift of spatial attention. One hypothesis suggested by the authors is that numbers convey the ordinal aspect more strongly than letters. Nonetheless, the dissociation observed in our study between numbers and letters provides further support for the view that only numbers, but not letters, impact the processing of spatial extent.

In the brightness-reproduction task, we observed no significant bias both when participants were cued with digits or letters. It could be argued that the flankers were not attended exactly the same way in the two tasks, because the brightness level of the first square could have been perceived by focusing only on the central part of the square. However, we believe both the short presentation time of the square to be reproduced and its relatively small spatial eccentricity on the screen make it unlikely that the flankers have been processed in a dramatically different way between the two tasks.

We can also hypothesize that, since the size of the second square was identical to the first one in the brightness task, focusing on a limited portion of the first square would have produced a global bias in the estimation of the brightness, which was not evident in our data. Many studies are pointing toward the idea that some continuous dimensions of magnitude share a more privileged relation, such as number, time and space. Brightness, while not usually described as being part of this group of dimensions, can elicit similar kinds of interference at the perceptual level (Xuan et al., 2007; Westheimer, 2008). In a recent review, Bonn and Cantlon (2012) described two main models to account for the links between the representational systems for multiple continuous dimensions of magnitude. On one hand, one can postulate the existence of an amodal (“adimensional,” as suggested by the authors) higher level of representation. On the other hand, the interference between dimensions can be interpreted as simple associations or conditional probabilities. While the former model suggests the unicity of a system of representation of magnitude, the latter points toward the existence of several systems of representation, with different degrees of overlapping between each of them. From this perspective, we believe our results bring new elements to the question of the links between distinct dimensions of magnitude.

Our results support the idea of privileged links between numbers and space (see also Pinel et al., 2004; de Hevia et al., 2012). First, the observed bias in size reproduction elicited by digits supports both the possibility of a shared representation of magnitude for numbers and spatial extent, and/or a privileged link between the two dimensions. Further studies are needed to test whether perceived duration is also impacted by the presentation of irrelevant magnitude information in the reproduction task, in order to confirm the privileged relation between number, space and time. Second, the absence of cognitive illusion when participants had to reproduce the brightness of the stimulus tends to indicate that this continuous dimension of magnitude does not share the same link. One possibility is that the underlying representational systems are dimension-dependent. It is possible indeed that while the representation of brightness can be associated with other dimensions such as size, its association with numbers is weaker or absent. As we mentioned in our introduction, the possibility of interference between numbers and luminance has been previously observed in a comparison task (Cohen Kadosh and Henik, 2006), although the association appears to be weak (Pinel et al., 2004). Also, neuroimaging techniques might prove more sensitive in disclosing a number-brightness interaction that is not revealed by behavioral methods (see for instance Ranzini et al., 2009).

Our results also fit with the model proposed by Pinel and colleagues concerning the neural correlates of the interference effects. They observe that the amount of overlap between the regions activated by the comparison of numbers, size and luminance, corresponds to (and predict) the amount of interference observed behaviorally between these dimensions. While numbers and size both show strong behavioral interference in comparative judgments and strong overlapping at the neural level, the association between number and luminance both at the behavioral and neural level is weaker. These results lead the authors to invoke a distribution of more or less overlapping neural systems of representations along the intraparietal sulcus. It is interesting, as the authors stress that these results show that the same interference effect on reaction times could arise from distinct neural coding. Our study supports the idea that, while an abstract level of representation could account for the observation of a cognitive illusion between numbers and space, a weaker association between the dimensions of numbers and brightness would make such interference harder to observe.

As we mentioned in the introduction, some authors argue for a functional locus of the number-space interactions at the response level (Keus and Schwarz, 2005; Gevers et al., 2006). We believe the paradigm used in our study allows addressing some aspects of that question.

First, some authors have suggested that the shared mechanism between dimensions consists in fact in a comparison system, rather than a representational one (Schwarz and Ischebeck, 2003; Cohen Kadosh et al., 2008b). While it could be argued that the reproduction task used here involves a comparison process (between the target square and the memorized square to be reproduced), the latter does not occur between the flankers given the absence of disparity between them. Second, one could argue that the effects observed in our study are due to a possible confound concerning the lateralization of the response keys assigned to the smaller/darker and larger/brighter responses the hypothesis of an interaction at the response level, in line with the hypothesis of an interaction occurring at the response level. However, we believe that the effects observed in our study are unlikely to be related to such confound. One reason is that the reproduction task, by asking the participants to maintain the characteristics of the first square in memory, pushes the comparison process to the response phase, i.e., after the presentation of the symbols. Also, the presence of unlimited time at the response stage might have diminished the likelihood that the activation of lateralized spatial response codes is responsible for the effects observed in the present study. In fact, it has been shown that the impact of numerical magnitude in the speeded lateralized manual and attentional tasks is restricted to specific short-timed temporal windows (Fischer et al., 2003; Mapelli et al., 2003). Moreover, such an effect of the lateralization of the response key should also have affected the results observed in the brightness-reproduction task, where no estimation biases have been found.

While we believe these aspects of the paradigm used in our study do not allow ruling out the possibility of an interaction occurring at the response level between numerical magnitude and spatial extent, further studies are needed to fully address the question of the functional locus of this interaction.

In summary, our results confirm the crucial role of numerical magnitude in eliciting a bias in the perception of other continuous dimensions such as spatial extent. Altogether, while we cannot rule out the possibility of a shared representation for dimensions sharing a privileged relation such as numbers and space, these results more generally support the idea of distinct but overlapping systems responsible for the interactions between different dimensions of magnitude, including brightness. While further studies are needed to test for the experimental contexts likely to elicit interference between brightness and number, our findings suggest that the underlying cognitive system is distinct from the one responsible for the number-size bias reported here.

### Conflict of interest statement

The authors declare that the research was conducted in the absence of any commercial or financial relationships that could be construed as a potential conflict of interest.

## References

[B1] Bonato M. Priftis K. Marenzi R. Zorzi M. (2008). Modulation of hemispatial neglect by directional and numerical cues in the line bisection task. Neuropsychologia 46, 426–433 10.1016/j.neuropsychologia.2007.08.019 17931670

[B2] Bonn C. D. Cantlon J. F. (2012). The origins and structure of quantitative concepts. Cogn. Neuropsychol. 29, 149–173 10.1080/02643294.2012.707122 22966853 PMC3894054

[B3] Brainard D. H. (1997). The psychophysics toolbox. Spat. Vis. 10, 443–446 10.1163/156856897X00357 9176952

[B4] Bueti D. Walsh V. (2009). The parietal cortex and the representation of time, space, number and other magnitudes. Philos. Trans. R Soc. Lond. B Biol. Sci. 364, 1831–1840 10.1098/rstb.2009.0028 19487186 PMC2685826

[B5] Calabria M. Rossetti Y. (2005). Interference between number processing and line bisection: a methodology. Neuropsychologia 43, 779–783 10.1016/j.neuropsychologia.2004.06.027 15721190

[B6] Casarotti M. Michielin M. Zorzi M. Umiltà C. (2007). Temporal order judgment reveals how number magnitude affects visuospatial attention. Cognition 102, 101–117 10.1016/j.cognition.2006.09.001 17046735

[B7] Cohen Kadosh R. Cohen Kadosh K. Henik A. (2008a). When brightness counts: the neuronal correlate of numerical–luminance interference. Cereb. Cortex 18, 337–343 10.1093/cercor/bhm058 17556772

[B9] Cohen Kadosh R. Lammertyn J. Izard V. (2008b). Are numbers special? An overview of chronometric, neuroimaging, developmental and comparative studies of magnitude representation. Prog. Neurobiol. 84, 132–147 10.1016/j.pneurobio.2007.11.001 18155348

[B8] Cohen Kadosh R. Henik A. (2006). A common representation for semantic and physical properties. Exp. Psychol. 53, 87–94 10.1027/1618-3169.53.2.87 16909932

[B10] Daar M. Pratt J. (2008). Digits affect actions: the SNARC effect and response selection. Cortex 44, 400–405 10.1016/j.cortex.2007.12.003 18387571

[B12] de Hevia M.-D. Girelli L. Bricolo E. Vallar G. (2008). The representational space of numerical magnitude: illusions of length. Q. J. Exp. Psychol. 61, 1496–1514 17924288 10.1080/17470210701560674

[B11] de Hevia M. D. Girelli L. Vallar G. (2006). Numbers and space: a cognitive illusion? Exp. Brain Res. 168, 254–264 10.1007/s00221-005-0084-0 16044296

[B13] de Hevia M.-D. Spelke E. S. (2009). Spontaneous mapping of number and space in adults and young children. Cognition 110, 198–207 10.1016/j.cognition.2008.11.003 19095223 PMC2705970

[B14] de Hevia M. D. Vanderslice M. Spelke E. S. (2012). Cross-dimensional mapping of number, length and brightness by preschool children. PLoS ONE 7:e35530 10.1371/journal.pone.0035530 22536399 PMC3334896

[B15] Dehaene S. (1992). Varieties of numerical abilities. Cognition 44, 1–42 10.1016/0010-0277(92)90049-N 1511583

[B16] Dehaene S. Bossini S. Giraux P. (1993). The mental representation of parity and number magnitude. J. Exp. Psychol. Gen. 122, 371–396 10.1037/0096-3445.122.3.371

[B17] Dehaene S. Piazza M. Pinel P. Cohen L. (2003). Three parietal circuits for number processing. Cogn. Neuropsychol. 20, 487–506 10.1080/02643290244000239 20957581

[B18] Dodd M. D. Van der Stigchel S. Leghari M. A. Fung G. Kingstone A. (2008). Attentional SNARC: there's something special about numbers (let us count the ways). Cognition 108, 810–818 10.1016/j.cognition.2008.04.006 18538756

[B19] Fias W. (2001). Two routes for the processing of verbal numbers: evidence from the SNARC effect. Psychol. Res. 65, 250–259 10.1007/s004260100065 11789429

[B20] Fias W. Brysbaert M. Geypens F. d'Ydewalle G. (1996). The importance of magnitude information innumerical processing: evidence from the SNARC effect. Math. Cogn. 2, 95–110

[B21] Fias W. Lammertyn J. Caessens B. Orban G. A. (2007). Processing of abstract ordinal knowledge in the horizontal segment of the intraparietal sulcus. J. Neurosci. 27, 8952–8956 10.1523/JNEUROSCI.2076-2007.2007 17699676 PMC6672167

[B22] Fias W. Lauwereyns J. Lammertyn J. (2001). Irrelevant digits affect feature-based attention depending on the overlap of neural circuits. Brain Res. Cogn. Brain Res. 12, 415–423 10.1016/S0926-6410(01)00078-7 11689301

[B23] Fischer M. H. (2001a). Cognition in the bisection task. Trends Cogn. Sci. 5, 460–462 10.1016/S1364-6613(00)01790-3 11684466

[B24] Fischer M. H. (2001b). Number processing induces spatial performance biases. Neurology 57, 822–826 10.1212/WNL.57.5.822 11552011

[B25] Fischer M. H. Castel A. D. Dodd M. D. Pratt J. (2003). Perceiving numbers causes spatial shifts of attention. Nat. Neurosci. 6, 555–556 10.1038/nn1066 12754517

[B26] Fischer M. H. Warlop N. Hill R. L. Fias W. (2004). Oculomotor bias induced by number perception. Exp. Psychol. 51, 91–97 10.1027/1618-3169.51.2.91 15114901

[B27] Galton F. (1880). Visualised numerals. Nature 21, 252–256 10.1038/021252a022800399

[B28] Gevers W. Ratinckx E. De Baene W. Fias W. (2006). Further evidence that the SNARC effect is processed along a dual-route architecture: evidence from the lateralized readiness potential. Exp. Psychol. 53, 58–68 10.1027/1618-3169.53.1.58 16610273

[B29] Gevers W. Reynvoet B. Fias W. (2003). The mental representation of ordinal sequences is spatially organized. Cognition 87, B87–B95 10.1016/S0010-0277(02)00234-2 12684205

[B30] Gevers W. Reynvoet B. Fias W. (2004). The mental representation of ordinal sequences is spatially organized: evidence from days of the week. Cortex 40, 171–172 10.1016/S0010-9452(08)70938-9 15174454

[B31] Gevers W. Santens S. Dhooge E. Chen Q. Van den Bossche L. Fias W. (2010). Verbal-spatial and visuospatial coding of number-space interactions. J. Exp. Psychol. Gen. 139, 180–190 10.1037/a0017688 20121318

[B32] Girelli L. Lucangeli D. Butterworth B. (2000). The development of automaticity in accessing number magnitude. J. Exp. Child Psychol. 76, 104–122 10788305 10.1006/jecp.2000.2564

[B33] Henik A. Tzelgov J. (1982). Is three greater than five: the relation between physical and semantic size in comparison tasks. Mem. Cognit. 10, 389–395 7132716 10.3758/bf03202431

[B34] Hubbard E. M. Piazza M. Pinel P. Dehaene S. (2005). Interactions between number and space in parietal cortex. Nat. Rev. Neurosci. 6, 435–448 15928716 10.1038/nrn1684

[B35] Jou J. Aldridge J. W. (1999). Memory representation of alphabetic position and interval information. J. Exp. Psychol. Learn. Mem.Cogn. 25, 680–701 10.1037/0278-7393.25.3.680 10368928

[B36] Keus I. M. Schwarz W. (2005). Searching for the functional locus of the SNARC effect: evidence for a response-related origin. Mem. Cogn. 33, 681–695 10.3758/BF03195335 16248333

[B37] Koten J. W. Jr. Lonnemann J. Willmes K. Knops A. (2011). Micro and macro pattern analyses of FMRI data support both early and late interaction of numerical and spatial information. Front. Hum. Neurosci. 5:115 10.3389/fnhum.2011.00115 22028688 PMC3199539

[B38] Loetscher T. Bockisch C. J. Nicholls M. E. R. Brugger P. (2010). Eye position predicts what number you have in mind. Curr. Biol. 20, R264–R265 10.1016/j.cub.2010.01.015 20334829

[B39] Mapelli D. Rusconi E. Umiltà C. (2003). The SNARC effect: an instance of the Simon effect? Cognition 88, B1–B10 12804817 10.1016/s0010-0277(03)00042-8

[B40] Perrone G. de Hevia M. D. Bricolo E. Girelli L. (2010). Numbers can move our hands: a spatial representation effect in digits handwriting. Exp. Brain Res. 205, 479–487 10.1007/s00221-010-2383-3 20700733

[B41] Pinel P. Piazza M. Le Bihan D. Dehaene S. (2004). Distributed and overlapping cerebral representations of number, size, and luminance during comparative judgments. Neuron 41, 983–993 10.1016/S0896-6273(04)00107-2 15046729

[B42] Previtali P. de Hevia M. D. Girelli L. (2010). Placing order in space: the SNARC effect in serial learning. Exp. Brain Res. 201, 599–605 10.1007/s00221-009-2063-3 19888566

[B44] Ranzini M. Dehaene S. Piazza M. Hubbard E. M. (2009). Neural mechanisms of attentional shifts due to irrelevant spatial and numerical cues. Neuropsychologia 47, 2615–2624 10.1016/j.neuropsychologia.2009.05.011 19465038

[B43] Ranzini M. Girelli L. (2012). Exploiting illusory effects to disclose similarities in numerical and luminance processing. Atten. Percept. Psychophys. 74, 1001–1008 10.3758/s13414-012-0302-3 22477059

[B45] Ren P. Nicholls M. E. R. Ma Y. Chen L. (2011). Size matters: non-numerical magnitude affects the spatial coding of response. PLoS ONE 6:e23553 10.1371/journal.pone.0023553 21853151 PMC3154948

[B46] Restle F. (1970). Speed of adding and comparing numbers. J. Exp. Psychol. 83(2 Pt 1), 274–278 10.1037/h002857321985457

[B47] Rusconi E. Kwan B. Giordano B. L. Umiltà C. Butterworth B. (2006). Spatial representation of pitch height: the SMARC effect. Cognition 99, 113–129 10.1016/j.cognition.2005.01.004 15925355

[B48] Santens S. Gevers W. (2008). The SNARC effect does not imply a mental number line. Cognition 108, 263–270 10.1016/j.cognition.2008.01.002 18313655

[B49] Santiago J. Lupiáñez J. Pérez E. Funes M. J. (2007). Time (also) flies from left to right. Psychon. Bull. Rev. 14, 512–516 17874598 10.3758/bf03194099

[B50] Schwarz W. Ischebeck A. (2003). On the relative speed account of number-size interference in comparative judgments of numerals. J. Exp. Psychol. Hum. Percept. Perform. 29, 507–522 10.1037/0096-1523.29.3.507 12848323

[B51] Uchikawa K. Ikeda M. (1986). Accuracy of memory for brightness of colored lights measured with successive comparison method. J. Opt. Soc. Am. A 3, 34–39 10.1364/JOSAA.3.000034 3950790

[B52] Van Dijck J.-P. Fias W. (2011). A working memory account for spatial–numerical associations. Cognition 119, 114–119 10.1016/j.cognition.2010.12.013 21262509

[B53] Van Opstal F. Gevers W. De Moor W. Verguts T. (2008). Dissecting the symbolic distance effect: comparison and priming effects in numerical and non-numerical orders. Psychon. Bull. Rev. 15, 419–425 10.3758/PBR.15.2.419 18488662

[B54] Vicario C. M. (2012). Perceiving numbers affects the internal random movements generator. ScientificWorldJournal 2012:347068 10.1100/2012/347068 22629133 PMC3353301

[B55] Walker P. Walker L. (2012). Size-brightness correspondence: crosstalk and congruity among dimensions of connotative meaning. Atten. Percept. Psychophys. 74, 1226–1240 10.3758/s13414-012-0297-9 22484796

[B56] Walsh V. (2003). A theory of magnitude: common cortical metrics of time, space and quantity. Trends Cogn. Sci. 7, 483–488 10.1016/j.tics.2003.09.002 14585444

[B57] Westheimer G. (2008). Illusions in the spatial sense of the eye: geometrical-optical illusions and the neural representation of space. Vision Res. 48, 2128–2142 10.1016/j.visres.2008.05.016 18606433

[B58] Xuan B. Zhang D. He S. Chen X. (2007). Larger stimuli are judged to last longer. J. Vis. 7, 21–2.5. 10.1167/7.10.2 17997671

